# Omics Insights into Animal Resilience and Stress Factors

**DOI:** 10.3390/ani11010047

**Published:** 2020-12-29

**Authors:** Federica Basile, Camilla Capaccia, Danilo Zampini, Tommaso Biagetti, Silvana Diverio, Gabriella Guelfi

**Affiliations:** 1 Department of Veterinary Medicine, Università degli Studi di Perugia, via San Costanzo 4, 0126 Perugia, Italy; fedee.basile98@gmail.com (F.B.); camilla.capaccia@gmail.com (C.C.); danilo.zampini@unipg.it (D.Z.); silvana.diverio@unipg.it (S.D.); 2 Departement of Economics, Università degli Studi di Perugia, via Alessandro Pascoli 20, 06123 Perugia, Italy; Tommaso.biagetti@libero.it

**Keywords:** animal resilience, environmental stressors, epigenetics

## Abstract

**Simple Summary:**

Resilience is the ability to adapt well in the face of adversity, trauma, tragedy, threats, or significant sources of stress. The concept of resilience has been applied to a wide range of systems ranging from cells to organisms. Environmental factors adaptively modulate resilience by intervening with transgenerationally transmitted epigenetic modifications. This review was organized to conceptualize the dynamic nature of molecular responses to stressors and the physiological role of resilience in maintaining or restoring normal homeostasis.

**Abstract:**

Resilience is conceived as a dynamic developmental process involving the achievement of positive adaptation within the context of significant adversity. Resilience is not a unique ability but rather a set of capacities of a system put in place to absorb a disturbance and to reorganize while trying to retain the same function, structure, and identity. This review describes the characteristics and the molecular mechanisms of resilience to understand the core elements of resilience and its indicators. The objectives of this review are: (1) to define some of the leading environmental stressors and clarify the mechanism of vulnerability or resilience outcomes; (2) to clarify some of the prominent epigenetic modulations mediating resilience or vulnerability as a stress response; (3) to highlight the neural mechanisms related to stress resilience since the central nervous system is a highly dynamic structure characterized by an everlasting plasticity feature, which therefore has the opportunity to modify resilience. The review aims to introduce the reader to the concept of resilience seen as an ability acquired in life and not only inherited from birth.

## 1. Understanding Resilience

**Figure 1:**
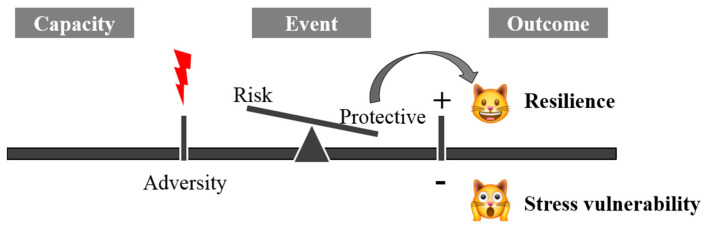
The mechanism of systemic resilience.

The term resilience is derived from the Latin word *resilire* (jump back, bounce), initially applied in physics to indicate the property of some materials to resist shocks without breaking or deforming. It was later used in medicine to indicate the ability to resist and react to adversity. Mammalian resilience is conceived as a dynamic, positive adaptation within a significantly adverse context [1]. Two critical conditions are implicit within this conceptualization of resilience: the exposure to severe adversity; and the acquisition of positive adaptation (Figure 1).

Stress is a severe adversity. The term stress is derived from the Latin word *stringere*, meaning to draw tight. Currently, the literature defines stress as a real or perceived perturbation of the physiological homeostasis or psychological well-being of an organism. To contrast the perturbation and return to normality, an organism uses various behavioral or physiological mechanism. Adaptation in the face of stress is a significant priority for all organisms.

The acquisition of positive adaptation is resilience. The resilience process can be based on three subsequent phases: disturbance, response, and outcome. Based on this theory, Döring et al. [2] defined resilience as the ability of a system, or an individual, to react (respond) to an external force (disturbance) while fulfilling some different conditions at the end of the response (outcome). The disturbance is identified as an external source to the system, leading to stress or resilience. The frequency and severity characterize the disturbance. The frequency of the disturbance is low when the disturbance manifests itself as unexpected or rare.

Conversely, the frequency is high when the disturbing events are repeated. The disturbance severity is difficult to quantify because it is closely related to the ability of organisms to adapt to the system. Disturbances can be ranked as macro- or micro-environmental factors. Macro-environmental factors are features associated with the environment and affect most subjects (e.g., disease pressure, ambient temperature): the response to these factors can be a genetic variance of the entire population. Micro-environmental factors are variations that concern only a minority of the whole population within that macro-environment (e.g., social interactions, nutrition). From a practical point of view, resilience to occasional macro-environmental disturbances-such as disease outbreaks and heatwaves-is less frequent and, therefore, less significant. Döring et al. claimed that the response (outcome) of the resilient system to disturbing events could lead the system to buffer, absorb, tolerate, cope, or adapt to the disturbances. Döring et al. claim that the response of the resilient system to disturbing events can lead the system to buffer, absorb, tolerate, cope, or adapt to the disturbances. The outcome falls within many definitions of resilience; on the one hand, definitions are required as a criterion of resilience, that the system responding to the disturbance succeeds, or at least maintains functionality. On the other hand, the requirement is that the system must neither change its structure nor collapse into a different state [2]. In 2014 the American Psychological Association defined resilience as “*the process of adapting well in the face of adversity, trauma, or significant sources of stress*” [3].

In 2015, the concept of “preclusion” defined resilience as “the ability of an individual to limit or preclude the detrimental effects of a stressor” [4]. That same year, resilience acquired multifaceted meanings, showing conceptual similarities with other notions such as adaptation, homeostasis, allostasis, and invulnerability. But these notions, although similar, show conceptual differences. The concept of adaptation is limited to a specific stress, but it does not, by itself, indicate flexibility in successful adaptation to all new challenges over a lifetime. While resilience allows the system to make significant changes, it is not limited to specific stress. The term homeostasis defines the self-regulatory capacity of living beings, which is very important to keep the internal environment constant despite variations in the external environment. However, while homeostasis is based on achieving a status quo because of feedback mechanisms, resilience, through dynamic and complex processes, allows active adaptation to new conditions. Allostasis is the ability to maintain the physiological stability of systems through change: this definition is very similar to the concept of resilience, but it does not focus on recovery after diseases [2].

Over the past decade, scientific studies on resilience in stressful situations have developed in many areas, especially in mammals, and resilience has been examined across a range of contexts, such as physiological (e.g., disease, temperature stress) or psychological (e.g., novel environment, social stressor, interaction). The new concept of resilience is a “general” combined trait, consisting of different resilience types depending on the nature of the disturbance [5].

To date, the research on animal resilience investigates mainly two aspects: animal productivity and animal welfare [6]. Resilience might be measured based on deviations from expected production levels over a period of time. However, detecting specific and sensible resilience indicators might provide the opportunity to include resilience in the breeding goals. The advantage of genetic selection, in contrast to management improvements, is that it affects all subsequent generations of livestock. Resilient animals are animals that need little/less attention time: increasing resilience is, therefore, desired.

Referred behavioral and physiological responses to stressful stimuli have been termed coping resources. Importantly, coping styles do not equate with success in coping with stressors [5]: the term describes the ongoing strategy or processes an animal employs when reacting to stressors rather than the coping response outcome [7]. Coping is a series of continually changing cognitive and behavioral efforts to manage specific external and internal demands. Coping strategies are essential to minimize the impact of stress and determine the degree of resilience or susceptibility. Coping is active when a subject tries to deal with a challenge, deals with fears, participates in problem-solving, and seeks social support. It also engages positive reassessment of aversive experiences that can produce long-term resilience.

In contrast, passive coping involves denial, avoidance of conflicts, suppressing emotions, and behavioral disengagement. It is maladaptive and provides only short-term resilience to stress [8]. Some differences were observed among animals between coping styles in susceptibility to stress-related diseases and when reacting to immunological stimuli [5]. Indeed, behavioral types can differ according to environmental changes, as demonstrated by the influence of audacity/shyness on grazing behavior in sheep [9].

From this perspective, in the current research topic, we aimed at collecting evidence from both animal models and human studies underlining the existence of similar strategies. An important aspect to highlight is how resilience is not a unique ability but rather a set of capacities of a system, put in place to absorb a disturbance and reorganize it while changing, to retain the same function, structure, and identity. Since resilience is not a unique ability, it is complicated to measure. For this reason, researchers prefer to define the characteristics through which resilience presents itself (animal behavioral) rather than directly measuring the resilience process. Another aspect to consider in measuring resilience is the different individual responses to the same stressor; faced with the same risk factor, some animals seem to be vulnerable, while others appear to be resilient. Each animal rates stressful situations differently, and the perceived level of stress varies greatly: this is why stressors are often categorized by their nature and not by their severity.

We conclude that stress, in the vulnerability models, provides a powerful tool for translational resilience research, and the animal model represents good support for understanding the intricate interplay between genome, behavior, and environment [10].

## 2. Stress Vulnerability and Resilience

Essential to understanding the precise meaning of resilience is to introduce the concept of stress because the features of stressful events define if and when an animal will go back to a pre-crisis status. Stress is defined as any factor of different nature (physical, chemical, behavioral, social) that changes or threatens homeostasis, thereby eliciting specific response mechanisms. Two contrasting hypotheses have explained the impact of stress. The cumulative stress models claim that the build-up of stress across the life span or adversity never has any beneficial effect; instead, the risk of disease gradually increases [11]. The match/mismatch model, which explains the concept of stress/epigenetic changes, includes adaptation to early-life stressors (even significant cumulative stressors) for specific individuals, thus including the concept of resilience. Early-life exposure to stressors can bring about epigenetic changes to match an organism to its environment and decrease the risk of vulnerability. A mismatch between the phenotypic outcome of the epigenetic changes and the ability to cope with current environmental stressors increases the risk of vulnerability [12].

**Figure 2:**
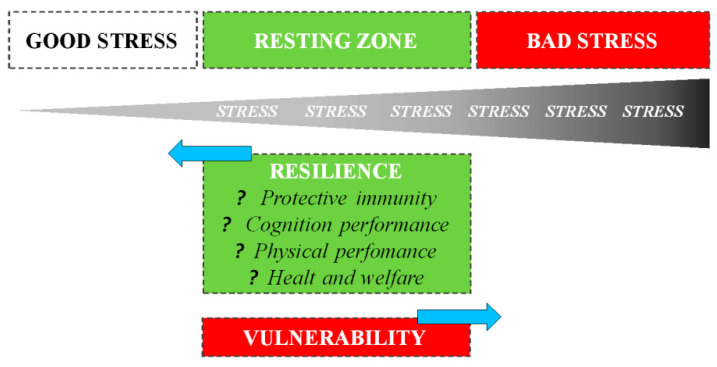
Staying on the good side of the stress spectrum to maintain health, one needs to optimize GOOD stress, maximize the RESTING ZONE, and minimize BAD stress. A resting zone exists between bad (harmful) and good (beneficial) stress, and the efficiency with which an organism returns to its resting zone following stress depends on its resilience.

The way an organism perceives and responds to stressors changes based on previous stress exposures: in general, exposure to recurring sources of stress induces chronic stress, associated with negative consequences for both physical and behavioral health. However, it is also essential to understand that the evolution process did not select the biological response to stress to harm or kill the animal, but rather improve survival [13]. A psycho-physiological stress response is one of the fundamental survival mechanisms of nature, e.g., without a fight-or-flight stress response, the gazelle has no chance of escaping, just as a lion has no chance of catching a gazelle [14]. Thus, during short-term stress, multiple physiological systems are activated to enable survival. Mild or moderate exposure to stress is much less likely to result in adverse health consequences: on the contrary, it may be beneficial to development [15]. Numerous studies have shown that both in humans and animals, short-term stress experienced at the time of immune activation induces a significant enhancement of the ensuing immune response [16,17]. Dhabhar et al. [13] first proposed that the short-term stress response prepares the cardiovascular, musculoskeletal, and neuroendocrine systems for fight or flight or prepare the brain for the challenges (e.g., figuring out an escape route) with a model of spectrum stress. On one side, we have good stress or eustress, which involves a rapid biological response mounted in the presence of the stressor, followed by a rapid, responsive shut-down on cessation of the stressor: such responses induce physiological conditions that are likely to enhance protective immunity, mental and physical performance, and overall health. The opposite end of the spectrum is characterized by bad stress or distress, which involves chronic or long-term biological changes that are likely to result in deregulation or suppression of immune function, a decrease in mental and physical performance, and an increased likelihood of disease: the deregulation of these latter processes persists long after the stressor has ceased (Figure 2).

The stress spectrum also describes the resting zone of low/no stress representing a state of health maintenance/restoration: after stressing, the amplitude, speed, and efficiency with which an organism returns to a resting state depends on its resilience.

To reconcile the differences between the cumulative stress model [18] that does not allow the subject to adapt to epigenetic changes and the match/mismatch model [12] that includes the concept of adaptation to early-life stressors, the three-hit concept of vulnerability and resilience arises [19]. This model underlines the importance of gene-environment interactions during critical phases of early life brain development to switch from vulnerability to resilience outcome. Daskalakis et al. [19] consider following the three-hit model: the interaction of genetic factors (Hit 1, epigenetic factors) with early life experiences (Hit 2, experiences) causes altered endocrine regulations and epigenetic changes during brain development, programming gene expression patterns relevant for an evolving phenotype. These programmed phenotypes have different susceptibility to challenges later in life (Hit 3, phenotype), promoting resilience or vulnerability. If the subjects are exposed to the latter type of later-life environment, they will develop the vulnerability phenotype, but when exposed to another type of later-life environment, the same phenotype will result in resilience outcomes [15].

Epigenetics modifications refer to relevant gene expression changes (with subsequent changes in cellular phenotype) that result from mechanisms other than from changes in DNA nucleotide sequence. Such modifications are induced by environmental events that directly allow the genome to adapt during delicate developmental periods and possibly to a lesser extent-in adulthood, leading to changes in gene expression and neural function [20]. In recent years, fostered by unprecedented biomolecular developments, a new way was conceived of considering the response of the animal to stress and adversity as an individual feature strategy resulting from the interaction between environmental signals and genome: the epigenome. Unlike the genome sequence, epigenome marks are less stable and can change in response to various environmental stimuli. However, epigenetic marks, sensitive to environmental exposure, transform the local chromatin environment, affect DNA accessibility, and regulate gene transcription or interfere in the mRNA translation through non-coding RNA. Epigenetic marks can disrupt regular gene expression and protein expression profiles [21]. Stress factors can impact the levels and the turnover of epigenetic factors either directly or indirectly. Whereas, with the same mechanism, protective factors ameliorate or alter the response of the subject to environmental stress. Examples of protective factors are maternal care, emotional relationships, and social support [22]. The protective factors that can increase a resilient behavior are contrasted by the risk factors representing those conditions, which increase the probability of experiencing a specific pathology. They can be linked to genetic factors and lifestyles, for example, obesity, illness, brief primary maternal care, and any other stressors [23].

## 3. Environmental Stressors and Gene Responses

The theoretical and methodological papers collected in this section provide different perspectives to understand better some of the main mechanisms of stress and the following resilience or vulnerability implications. In mammals, the resilience system evolved as a stress-solving mechanism to enhance the survival of the animals. The resilience of the whole organism depends on the resilience of the subsystems regulating vital parameters and mood. More specifically, the continuous regulatory processes that balance stress between the subject and the environment result in a stable life development [24]. If systemic resilience decreases, the risks of morbidity and mortality increase. To best explore the interactions between environmental stressors and resilience, some potential stressful environmental sources, such as “sunlight, temperature, feeding, early-life conditions, social interactions [25].

### 3.1. Sunlight

**Figure 3:**
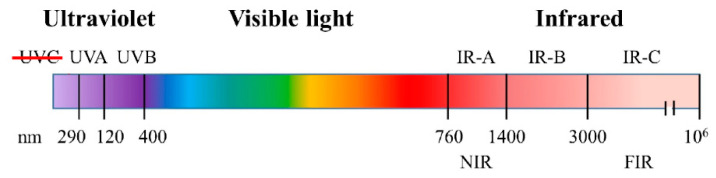
Solar spectrum composition. Red bar over UVC means that the ozone layer blocks them (NIR: near infrared, FIR: far infrared).

Solar radiation provides the Earth with light and heat; this radiant energy is necessary for the inhabitants of biological environments. The solar spectrum that reaches the surface of Earth consists of three bands: ultraviolet (UV), which makes up to 8% (290–400 nm); visible light (VL), up to 42.3% (390–700 nm); and infrared (IR), that reaches 49.4% (600–1000 nm), of the whole radiation. Each of these bands has a different impact on humans and animals (Figure 3). 

Overexposure to ultraviolet (UV) irradiation causes oxidative stress, inflammation, hyperpigmentation, mutation, and degradation of the extracellular matrix (ECM), resulting in wrinkling (disruption of hydrolipidic balance) and skin cancer [26]. UV-B radiation directly causes DNA mutations; UV-A predominantly acts indirectly via reactive oxygen species (ROS) generation in lipid rafts cell membranes.

**Figure 4:**
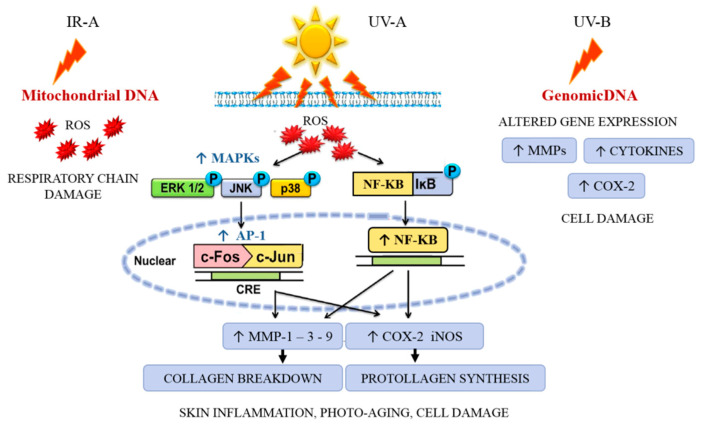
Sunlight UVB-induced inflammatory and photodamage.

The infrared waveband is divided into three bands: IR-A (760–1400 nm), IR-B (1400–3000 nm), and IR-C (3000 nm–1 mm). IR radiation can penetrate the epidermis, dermis, and subcutaneous tissue to differing extents depending on the exact wavelength range being studied. Exposure to IR is perceived as heat. IR-A is mainly absorbed in mitochondria and impairs the respiratory chain, which also results in ROS generation. ROS intervenes by driving the activation of mitogen-activated protein kinase MAPK (i.e., ERK, JNK, and p38) through recruiting the transcription factor activator protein-1alias AP-1 (c-Fos and c-Jun) to the nucleus and subsequently activating NF-κB for upregulating the pro-inflammatory gene expression [27,28]. The increase of AP-1 expression upregulates the gene and protein expression of MMPs [29] (especially MMP-1, MMP-3, and MMP-9) that degrade the ECM (e.g., collagen and elastin), causing coarse wrinkles and sagging skin, and characteristic inflammation signs (erythema) mediated by COX-2 and iNOS (Figure 4).

If, on the one hand, sunlight represents a stress factor capable of causing severe damage, on the other hand, moderate sunlight infrared (IR) exposures increase the synthesis of serotonin and melatonin, molecules involved in mood and animal cognition, suggesting that cognitive function may also be influenced by light. It is believed that serotonin and melatonin have evolved initially to provide molecular protection against free radicals [30]. Furthermore, sunlight allows the synthesis of vitamin D3 from the skin, which is essential in controlling blood calcium levels and maintaining muscle strength.

Another life stressor paradigm is that sunlight overexposure induces cortisol production [31]. Cortisol is a hormone that causes a cascade of physiological responses to maintain homeostasis, mediating the stress response. The eustress, or good stress, is required because an animal would not feel alert and ready to hunt without stress. Therefore, during stress, normal cortisol secretion could be read as a resilience signal to stress.

Research about the effect of light resumed in the 1960s, subsequently discovering new light sources, lasers, and LEDs, making it possible to precisely adjust the wavelength and radiation energy. In the following years, it was possible to demonstrate that high-intensity UV exposure has toxic effects on the skin, while low intensities induced vitamin D synthesis and tryptamine production, improving mood [32,33,34]. Based on these results, many researchers investigated the effect of IR light generated by laser sources on stress resilience regulation with great interest. In a review by Ferraresi et al. [35], the impact of IR light on skin resilience, supernormal muscle performance, and accelerated recovery of fatigue and injury. Research carried out on two groups of mice, one raised in complete darkness and the other with stressful light exposures, revealed that only the group exposed to light showed a retinal tissue with high light resilience levels [36]. The endogenous neurotrophic factors protected the photoreceptors from degenerating [37], and these findings led to the hypothesis that the retina responds to injury by upregulating neurotrophic factors to protect retinal cells, accelerating repair and wound healing. Consistent with this hypothesis are the observations that retinal bFGF and CNTF are elevated in animals undergoing light damage. Despite the upregulation of neurotrophic factors, severe loss of photoreceptors occurs in light-induced retinal degeneration, although not all researchers believe that these endogenous factors protect photoreceptors. One possible explanation is that these factors save photoreceptors because, in their absence, photoreceptor loss would be more severe.

### 3.2. Hot and Cold Temperature

Thermotolerance is a process that involves increased resilience to heat-induced stress. In isolated cells and animal studies, thermotolerance (also called heat preconditioning) is characterized by the increased resilience to heat stress that otherwise would be lethal. Thermotolerance has been divided into three phases: the induction, triggered by treatment at high temperatures; the development, which takes place under appropriate environmental conditions (e.g., temperature, pH); and the decay, which leads to complete thermotolerance disappearance [38].

The exposure of mice to whole-body hyperthermia offers heat-protection to subsequent exposures (41 °C for 40 min) [39]. Small ruminants show adaptive capacities determined by various mechanisms such as morphological, anatomical, behavioral, physiological, biochemical, and molecular characteristics that help animals survive in a specific environment. Goats are exposed to heat-induced stress to enhance heat resilience: they seek shade, reduce food intake, and increase water intake to dissipate heat via the evaporation process, both through the skin and the respiratory tract [40,41]. Analyzing the metabolism of ruminants subjected to thermal stress revealed that animals respond with a reduction in serum and plasma concentrations of thyroid hormones such as triiodothyronine (T3) and thyroxine (T4), in order to limit the basal metabolism to produce less metabolic heat and to adapt to heat stress challenges [42]. Non-esterified fatty acids (NEFA) are another critical metabolic regulator, whose reduced levels coincide with an increase in lipid absorption by the liver, which is very active during thermal stress [43]. Advances in molecular techniques allow for a better understanding of the molecular pathway involved in small ruminant thermo-tolerance: extending the knowledge of thermo-tolerance genes will enable a selection of ruminants with a high thermal shock resilience. It has been known since 1999 that the PIK3R3 gene regulated the growth of small ruminants, noticing that small tropical ruminants had a reduced body size, allowing them a low-quality pasture feeding ability and better thermoregulation [44]. BMP7, MSTN (GDF8), and STIL genes regulate the resilient phenotype in Egyptian sheep exposed to desert environmental conditions [45].

Over the years, thermal stress has been extensively studied in many ruminant species, as it is significant for reproductive efficiency. Thermal stress is regulated by the hypothalamic-pituitary-adrenal (HPA) axis, which in turn involves the secretion of glucocorticoids and catecholamines, the main inhibitors of the reproductive axis [46]. Temperature stress reduces the pituitary expression of FSH and FSRH, compromising ovarian follicular development [47]. A significant reduction in LH receptor genes (LHR) during heat-induced stress was observed in Shiba goats: this down-regulation of the LHR gene could be due to the reduced steroidogenic activity [40,48].

**Figure 5:**
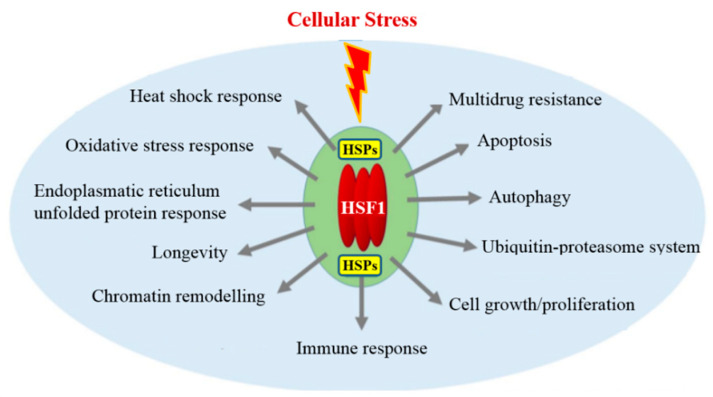
Heat shock factor 1 (HSF1) cellular stress response. HSF1 plays a central role in the upregulations of heat shock proteins (HSPs). HSF1 activity then upregulates vital components of diverse stress response pathways and processes.

In mammalian cells, Heat Shock Proteins (HSPs) are involved in the acquisition, maintenance, and thermal resilience decay. This family of proteins, named according to their molecular weight HSP60, HSP70, HSP90, is produced by cells in response to heat [49] cold [50], UV light [51], and wound healing or tissue remodeling [52]. HSPs, or stress proteins, are highly conserved and present in all cells of all organisms. A hypothermic transient stress induction in rat hepatoma cells activates the synthesis of HSP proteins with different molecular weights (60, 70, and 90 kDa) and related different thermotolerance levels. The HSPs induction and the maximum thermotolerance development occurs after 6–8 h from exposure to high temperatures; furthermore, the maintenance and degradation of HSPs levels are correlated to maintenance and decay of the thermotolerance. HSPs are upregulated in response to heat and a series of stressors such as plant toxins, caloric restriction, hypoxia, and exercise. HSPs are tissue protective: many members of this group perform chaperone functions by stabilizing new proteins to ensure correct folding or refolding of proteins damaged by cell stress. Various stress factors, leading to protein damage, induce a protective heat shock response to maintain eukaryotic protein homeostasis. Heat shock factor 1 (HSF1) plays a central role in the upregulations of HSPs gene expression. HSF1 acts in diverse stress-induced cellular processes and molecular mechanisms, and it emerges as a principal orchestrator of cellular stress response pathways [53] (Figure 5).

It is not yet clear whether exposure to moderate levels of cold induces tissue resilience. In general, the cellular effects of hypothermia include oxygen consumption, and metabolic rates decrease modifications in redox states and alterations in gene expression [54,55]. The molecular mechanism that regulates adaptation to mild cold stress is poorly understood, and it is probably brought about by a variety of different pathways, depending on the cellular situations. In moderate exposure to hypothermia, cold-shock proteins intervene, which guarantees cell adaptation to new environmental conditions, ensuring that the translation of specific mRNAs occurs at temperatures below physiological temperatures and providing resilience to the organism [56].

Cold-inducible RNA-binding protein (CIRP) and a cold-shock protein RBM3 (RNA-binding motif protein 3) are two RNA-binding proteins transcriptionally upregulated in response to a low temperature [57]. CIRP, like HSPs, acts as a molecular chaperone, maintaining the RNAs stability in response to thermal stress. CIRP and RNM3 regulate gene expression at the translational level by binding to the 5′-UTR or 3′-UTR region of mRNA and influencing the translation initiation speed or the stability of mRNA. During cellular stress, protein synthesis is severely reduced due to the impairment of the initiation step of mRNA translation [58]. CIRP accumulates in stress granules upon exposure: these are structures where translationally silent mRNAs accumulate in response to metabolic events in the cell [59]. It is known that RNA binding proteins, such as CIRP, regulate and aggregate specific mRNAs into stress granules [60,61]. CIRP binds specifically to the 3′-untranslated regions (3′-UTRs) of stress-responsive transcripts Replication Protein A2 (RPA2) gene and thioredoxin (TXN): both are proteins associated with increased survival after stress. TRX is a ubiquitous, multifunctional protein that regulates cellular signaling by quenching ROS: in its reduced form, TRX favors hydrogen peroxide conversion (H_2_O_2_, a strong oxidant) into H_2_O. CIRP and RBM3 seem to play a role in cell proliferation and cell transformation. It has been suggested that overexpression of CIRP may be responsible for impaired growth of the mammalian cell cycle at sub physiological temperatures with prolongation of the G1 phase of the cell cycle [62].

Moreover, CIRP plays a vital role in protecting cells against apoptosis [63]. Direct binding of RBM3 to mRNA might alter the secondary structure of mRNA in ways that affect both the access of initiation factors and ribosomal subunits and potentially the activation of kinases that regulate them. Overall, some translation-initiation proteins are associated with cap-dependent regulation and, although CIRP is also involved in cap-independent translation upon moderate cold-shock, CIRP and RBM3 might stimulate proliferation mainly by facilitating the cap-dependent mechanism at physiological temperatures [59].

### 3.3. Lack of Oxygen

Cellular and systemic oxygen homeostasis is a finely regulated process essential for energy metabolism and survival of mammalian cells. Mammals require a continuous supply of oxygen as they are obligate aerobes. Oxygen is distributed to the tissues through the pulmonary and systemic circulation and cannot be stored. Therefore, obligate aerobes need proper oxygen supply to prevent interruptions/low oxygen levels in the blood (ischemia).

**Figure 6:**
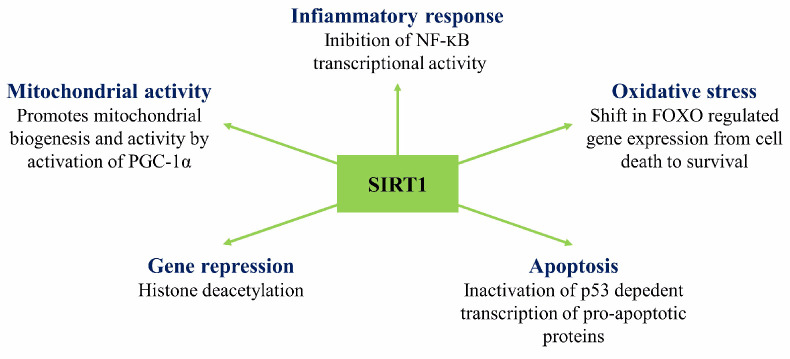
Nuclear activities of SIRT1. The regulation of gene expression by SIRT1 deacetyltransferase activity allows for the activation and inhibition of signaling pathways involved in numerous cellular functions.

The experimental efforts geared towards the study of ischemia show that prolonged ischemia causes the death of the affected tissue, while short-lived or partial ischemia induces tissue resilience as the tissue survives and reacts in self-protective ways [25]. The early studies on the protective effect of transient ischemia were developed by Murry et al. [64] through the coronary artery occlusion model in the heart of a dog. Murry et al. noticed that a single and brief episode of myocardial ischemia, like 5–15 min, may significantly impair the muscle, reducing ATP production. The metabolic and functional abnormalities generated may persist for hours or days. In ischemia, a significant cause of ATP degradation is the squandering of ATP by the mitochondrial ATPase. The rate of ATP degradation is regulated by ATPase inhibitor activity which binds the enzyme during ischemia-induced mitochondrial acidosis. In repeated episodes of ischemia, the inhibition of ATP degradation occurs more quickly through the instantaneous expression of Heat Shock Proteins, the result of a cardiac adaptation to ischemia. Murry et al. proposed that multiple anginal episodes that often precede myocardial infarction might protect the heart from a subsequent sustained ischemic insult [64]. This observation, therefore, implied that sublethal ischemia was inducing muscle resistance. Even at the molecular level, studies on ischemic conditioning have been done since 1998 [65]. Barone et al. [65], observed that, during the conditioning of brain ischemia in rats, the expressions of interleukin-1 receptor antagonist mRNA and protein expression increased, while c-fos mRNA expression was reduced. The effect of sublethal ischemia on retina HSP27, HSP70, and HSP90 signaled a marked increase in HSP27 mRNA and protein expression. A genome-wide association study of global expression profiles during the process of ischemic cell death and ischemic tolerance in the brain of the rat is necessary for a better understanding of the molecular pathophysiology of ischemia [66]. The hippocampal neurons can acquire resistance to ischemia when subjected to sublethal ischemia several days before lethal ischemia. The ischemic tolerance was associated with transient up-regulation of transcription factors (c-Fos, JunB Egr-1, -2, -4, NGFI-B), Hsp70, and MAP kinase cascade-related genes (MKP-1), implicated in cell survival [67]. A family of genes involved in resilient effects of mitochondrial function and cellular metabolism necessary for lethal cerebral ischemia protection are Sirtuins [68]. These stress-responsive enzymes can be linked to the process of modulating protective pathways against oxidative stress, energy depletion, DNA damage, and apoptosis (Figure 6).

Sirtuin 1 (SIRT1), is considered anti-ageing protein modulating protective mitochondrial pathways against oxidative stress. Transgenic mice overexpressing SIRT1 display an enhanced mitochondrial activity, defending against oxidative stress, DNA damage, and apoptosis. Besides, caloric restriction enhances SIRT1 activity, resulting in decreased ROS, preserved mitochondrial function, and enhanced signaling pathway of insulin growth factor, contributing to prolonged lifespan and ischemic protection shown in many invertebrate and vertebrate species.

Brooks et al. [69], highlight the release of cytokines from briefly ischemic heart muscles and their role in activating a cascade of intracellular pathways by activating the G protein-membrane receptors. The generated proteins converge on the internal mitochondrial membrane reducing the ROS production. The endogenous cytokines mediate the short-term protective response and activate nuclear transcription factors (NF-kB, AP-1, and HIP1a), triggering the synthesis of known tissue protection mediators (iNOS, COX-2, aldose reductase, HSP, and Mn-SOD). The ischemic conditioning, when delivered either before or after the ischemic event, can provide considerable cardioprotection. Hypoxia in adult rats increased hippocampal neurogenesis and, when administered after experimentally induced stroke, mitigated memory loss [25]. Hypoxia preconditioning (exposure to hypoxia before an experimental stroke) mitigated structural loss, reducing the infarct size by as much as 50%. These effects were associated with the expression of Hypoxia-induced expression of HIF-1α (hypoxia-inducible factor-1) and its target genes (erythropoietin, vascular endothelial-like growth factor) [25]. In all nucleated mammalian cells, heterodimeric transcription factor HIF-1 functions as the primary regulator of oxygen homeostasis [70]. The HIF-1α transcription factor, together with the SIRT1, act as oxygen and redox sensors, respectively. Lim et al. [71] suggested that cross-talk between oxygen- and redox-responsive signal transducers occur through the SIRT1-HIF-1alpha interaction.

### 3.4. Effects of Environmental Enrichment: Feeding and Early Life Conditions

Environmental enrichment is often used to improve animal welfare. The influence of enrichment may depend on the early and current life housing experience of animals. It is known that factors such as early life experiences, feeding, and the neonatal environment affect animal behavior, welfare, and resilience towards stressful situations throughout life. Guelfi et al. [72] demonstrated that feeding dogs enriched drug detection and improved stress resilience while dogs searched for drugs. Complementary feeding in detection dogs influences the expression of blood-cell neuroplasticity related genes, modifies systemic metabolism, and to the end, metabolic cross-talk increases the resilience of dogs during the drug detection. Complementary feeding (containing branched-chain and limiting amino acids, carnitine, vitamins, octacosanol) improves the physical fitness of working dogs by exerting beneficial effects on heart rate recovery time, energy metabolism, and biomarkers of muscle damage, suggesting that dietary supplements can increase resilience in the dog to cope with stressful work activities [73]. However, it is not easy to delineate the exact effects of nutrients or bioactive food components in all epigenetic modulations because nutrients also interact with genes, other nutrients, and different lifestyle factors.

Furthermore, every epigenetic phenomenon also interacts with cellular signaling, and cell-fate determination, adding complexity to the system and, possibly, complementary feeding affected epigenetic mechanisms at multiple levels [74]. Nutrients act as a source of methyl groups or co-enzymes for one-carbon metabolism-regulating methyl transfer [75]. Otherwise, nutrients and bioactive food components directly affect enzymes that catalyze DNA methylation and histone modifications [76]. Lastly, the diet is the input determining systemic metabolism, which modifies cellular milieu leading to epigenetic pattern changes [77].

Early life conditions, enriched environments, and personal characteristics (coping) are fundamental in the behavior and welfare of pigs. Intensive pig husbandry is poor of stimuli with limited possibilities of species-specific behaviors like less game behavior or less exploratory activities (e.g., rooting and chewing). These restrictions lead to maladaptive oral behaviors, and these pigs become much more biting than pigs living in enriched environments [78]. In intensive farms, for economic reasons, piglets are early-weaned (15- to 21-day weaning age). Early-weaning causes inexperience with solid foods, so piglets feed less, grow less, weigh less, and show reduced welfare [79]. Piglets subjected to the stress of premature maternal separation may show food neophobia or pathologies of the gastro-intestinal tract with modification of motility and gastric emptying and increasing diarrhea occurrence [78]. Whereas the piglets eating together with the sow have a positive eating behavior development and adapt well to being weaned [79].

In mice, breeding in an enriched environment (toys, a running wheel, a hut, tunnels, different height levels in the cage, or increased cage size) increases behavioral flexibility and resilience towards stressful situations; at the same time, it decreases the fear of contact with new objects and environments [80].

In neonatal poultry early, supplemental feeding given on the day of hatch and the two subsequent days stimulated the immune system development by providing improved resistance to disease challenge. Development of the immune system is initiated during embryogenesis but is not complete until weeks or months after hatching. This development may be limited by nutrient availability in fasted hatchlings [81]. Along with nutrition, various factors like temperature, air quality, light regime, and housing may act as stressors. These stressors can modify a poultry immune system and, therefore, affect vaccination response or disease susceptibility. The housing forms for poultry and birds differ mainly in the freedom of movement; indoor/outdoor loose housing where they can express their natural behavior and in cage systems. Loose housing is the prerequisite for high productivity, health, and welfare [82]. Research findings point that the innate immune system of loose housing poultry has stronger responsiveness [83,84,85,86]. In conclusion, early life feeding strategy and housing conditions influence poultry resilience to an immune challenge later in life. 

### 3.5. Social Interactions and Social Defeat

**Figure 7:**
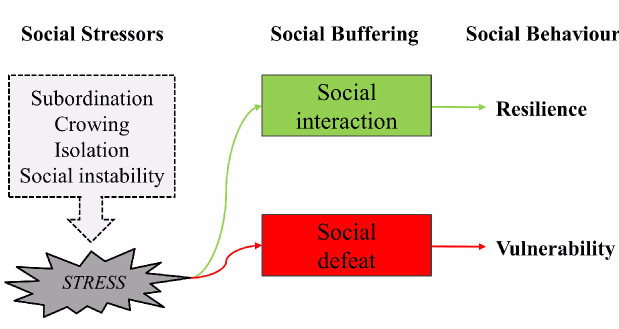
Social Interactions and Social Defeat.

In their lifetime, animals perform many activities aimed at survival and reproduction; for example, they find food and mates, defend themselves and, in many cases, care for their offspring or other relatives. These activities become “social” when they involve interactions among members of the same species in a way that influences immediate or future behavior [87]. Social interactions between organisms living in a group can positively and negatively affect characteristics such as their well-being, productivity, health, and individual resilience. In rodents, humans, and non-human primates, social interaction or social defeat has been shown to curb genetic and environmental vulnerabilities and confer resilience to stressors, probably via its effects on the hypothalamic-pituitary-adrenocortical system, the noradrenergic system, and central oxytocin pathways [88] (Figure 7).

Social interactions could depend on early life experiences, cooperation, and maternal behavior, all of which are examples of positive social interactions. At the same time, competition and aggression are examples of negative social interactions. In laying chickens, the incubation and rearing conditions substantially influence the incidence of feather pecking and cannibalism [89]. In pigs, early life isolation changed their behavior, neuroendocrine and immune regulation, with negative consequences for their health and well-being later in life [90]. Social interactions have a high impact on the phenotype and influence evolutionary processes and domestic breeding programs [89]. For example, cannibalism in laying hens depends on the genetic effect of the actor and social interactions originating from the victim [91].

Whole-genome sequencing can identify genetic variants associated with social interactions and social defeat using omics technologies (genomics, epigenomics, proteomics, metabolomics), proving that social interactions can impact brain gene expression, brain development, and behavior [87]. Gene transcripts from the brains of rats highlighted an active or a passive coping strategy in the face of social defeat induced stress, based on the latency, to exhibit a submissive posture. Differential expression analysis discovered quantitative changes in expression levels of a panel of 88 genes related to G protein-coupled receptor signaling. IL-1β was the only transcript found to be upregulated in passive coping animals and downregulated in active coping animals [92].

The application of genetic improvement programs aimed at the selection of animals with socially positive behaviors will help to simultaneously improve the welfare and productivity of livestock [89]. Recent studies suggest that vertebrate gene forkhead box P2 (foxp2) variation has critical social roles in animal communication and the development of socially embedded behaviors [93,94].

Social defeat is a concept used to study the physiological and behavioral effects of hostile interactions between conspecific animals in a group-individual context, potentially generating very significant consequences in terms of control over resources, access to mates, and social positions. An animal model, widely used in social defeat studies, requires a mouse resident in an experimental cage, which has established its territory, and the intruder subsequently placed in the cage [95]. There is a decrease in social interaction with unknown conspecific males in mating behavior and aggression. Defeated animals show decreased locomotor and exploratory activity, speed of movement, food, water intake reduction, and a grooming decrement. Animals who underwent social defeat show the stimulation of the HPA axis and increased blood level adrenocorticotropin hormone, adrenaline, and norepinephrine [95]. Defeat and subordination lower the levels of luteinizing hormone, follicle-stimulating hormone, and testosterone [95]. Consequently, the decline in androgen production alters the normal male stimuli, such as the production of preputial pheromones dependent on androgens, which constitutes the attack stimulus.

## 4. Resilience in Response to Epigenetics Remodeling

Nature (genetics) and nurture (life experience) through genetic and epigenetic mechanisms control the gene expressions in order to imprint a response of susceptibility or resilience to stress [8]. The term epigenetics derives from the Greek prefix “epi” which means upon or over, and genetics, which refers to the sequence of genomic DNA. Epigenetic transcriptional regulatory mechanisms include DNA methylation, post-translational modifications of histones, and changes in the position of the secondary structures formed by DNA and histones called nucleosomes, all of which are collectively referred to as the epigenome [96]. Another epigenetic mechanism includes non-coding R.N.A. (ncRNA) such as microRNA (miRNA) and large non-coding R.N.A. (lncRNA) [97]. LncRNAs are associated with chromatin modification complexes such as repressive complexes, recruiting and targeting them in specific genomic regions. These mechanisms can act separately or in synergy to modulate chromatin structure and its accessibility to the transcriptional machinery. Epigenetic mechanisms are highly dynamic and can be influenced by environmental factors such as diet, social/familial relationships, and stress [8].

The epigenome has the potential to encode a molecular memory of past events that can influence gene expression, neuronal function, and future behaviors. Epigenetic regulation of chromatin plays a role in determining the adaptive or maladaptive nature of neural and behavioral responses to environmental stressors [96]. Animal models of the epigenetics of stress responses have been most valuable for establishing the molecular and cellular mechanisms by which stressor-induced changes in chromatin regulation impact behavioral stress responses [96]. Stressors are among the environmental stimuli that can change DNA methylation patterns in the brain, and various stress paradigms have shown that they decrease methylation of multiple genes encoding intermediaries in the HPA axis. Neural plasticity genes, such as the BDNF, are also targets of regulation by DNA methylation [96].

For example, in rodents, the axis reactivity is reduced following high maternal care or a short adult separation, while the hyperactivity of the axis is associated with prenatal stress or prolonged maternal separation during growth [96]. The homeostatic regulation of the HPA axis is also affected by epigenetic regulation and DNA methylation of its multiple genes. A newborn rat exposure to a rodent abuse model with daily disruptive caregiving during the first postnatal week is associated with an increase in DNA methylation of BDNF exon IV and IX. BDNF methylation led to a down-regulating of the corresponding mRNA [96]. On the other hand, the exposure of adult rats to predator stress and social instability leads to increased DNA methylation and decreased mRNA expression of BDNF in the hippocampus, but not in the prefrontal cortex [98]. The latter suggests that BDNF methylation changes induced by environmental stress could contribute to brain plasticity and determine different behavioral responses to stressors [96]. In the presence of environmental stressors, the methylation or demethylation of DNA cytosine occurs through writers and erasers of enzyme regulation. Many of the proteins regulating methylation processes are subjected to stress-dependent expression changes as the Gadd45 scaffold family, the Aid/APOBEC family of cytosine deaminase, the methyl-DNA binding protein Mbd4, and the Tet protein family [96]. In mice, ten days of chronic social defeat stress caused a significant increase of DNMT3A mRNA expression in N-acetylcysteine to stimulate depressive behavior [99] which in turn gave rise, in the hypothalamic paraventricular nucleus (PVN), to the reduction of the Corticotrophin-Releasing Factor (CRF) promoter gene methylation [100]. CRF secreted from PVN neurons is a crucial regulator of the HPA axis after chronic social defeat stress exposure, and DNA methylation induction has a pro-depressive function.

DNA methylation was also associated with resilience to a different experimental stressor paradigm called chronic ultra-mild stress (CUMS). CUMS is induced by a series of mild environmental and social stressors that bring about depressive-like behaviors if protracted over time. Interestingly, after CUMS induction, susceptible and stress-resilient mouse strains showed an enhancement of methylation Glial-Derived Neurotrophic Factor (GDNF) promoter gene. GDNF gene DNA methylation is correlated with animal behavior. Susceptible strain methylation increase was associated with GDNF expression decrease. Whereas, in resistant strain methylation, the increase was associated with GDNF mRNA increase. This strain difference appears to arise from various proteins recruited to promoter methylation sites [100].

**Figure 8:**
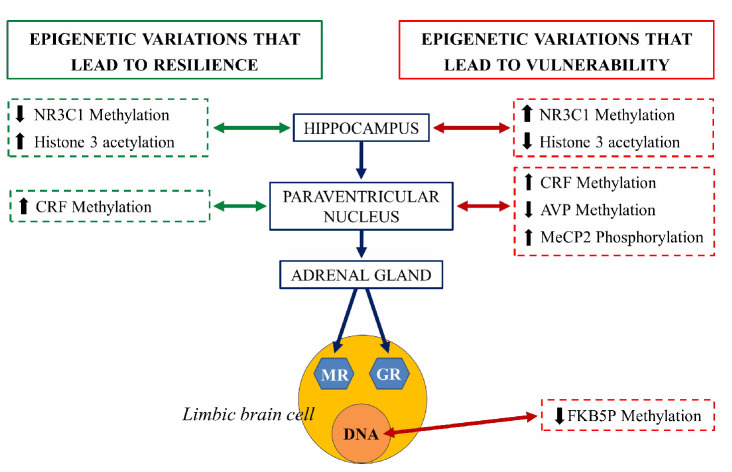
Schematic representation of the epigenetic regulation of the hypothalamus-hypophysis-adrenal axis (grey axis). The right side of the figure illustrates the epigenetic changes that lead to vulnerability to stress and risk at each structure (red arrow). The left side represents epigenetic variations that lead to resilience at each structure (green arrow). NR3C1: steroid receptor gene, pMeCP2: phosphorylated protein related to methylation of histones, CRF, corticotropin-releasing factor gene, AVP: arginine vasopressin gene, FKBP5: gene coding for chaperones for the expression of glucocorticoid receptors (GR) and mineralocorticoid receptors (MR) [104].

An extensively studied genetic target, subjected to epigenetic regulation, is the NR3C1 gene encoding glucocorticoid receptor (GR). Evidence suggests that the methylation of the NR3C1 promoter regions are related to vulnerability or stress resilience [101]. Hippocampal NR3C1 is upregulated in rats exposed to high maternal care during the early postnatal days: this mediates enhanced glucocorticoid feedback, long term decreased HPA axis responsivity and stress-resilient phenotypes during adulthood. In contrast, early life stress or low maternal care decreases hippocampal NR3C1 expression, increases HPA responsivity, and predisposes adults to stress vulnerable phenotypes. In both cases, the mRNA expression encoding the GR is inversely correlated with DNA methylation of CpG residues in the NR3C1promoter regions [96]. NR3C1 serves as a binding site for the transcription of nerve growth factor-inducible protein A (NGFI-A). During a critical period in the first week of life, high maternal care is thought to determine the set-point of HPA axis responsivity in adulthood through NR3C1 promoter demethylation that permits NGFI-A-dependent GR expression [102,103] (Figure 8).

Adverse early-life stress (ELS) induces, in mice, long-lasting alterations in passive stress coping and memory. This phenotype was accompanied by a persistent increase in the HPA neurons of arginine vasopressin (AVP) expression associated with DNA hypomethylation of methyl CpG-binding protein 2 (MeCP2) epigenetic marking. Thus, ELS controls DNA methylation in neurons to generate stable changes in AVP expression, which triggers neuroendocrine and behavioral alterations, features that are frequently identified in depression [105].

Recent studies on transgenerational inheritance in mammals suggest potential inheritance of epigenetic patterns via multiple mechanisms conferred by paternal sperm and maternal germline, potentially reflecting ancestral stress and impacting anxiety-related mental health in offspring by shaping endocrine programming, brain development, and ways to cope with stress [106]. Perturbed maternal behaviors by unpredictable separation and maternal stress widely affect methylation in the brain and cause hypomethylation or hypermethylation in the offspring of different genes altering the gene expression. Strikingly, the aberrant methylation is perpetuated across successive generations and is present in the germline of first-generation males and the brain and germline of second-generation progeny: this progeny and the following show multiple stress-related symptoms such as depressive-like behaviors and social anxiety. Due to disrupted maternal care, aberrant DNA methylation affects several tissues; it can subsist after meiosis in male germ cells and is trans-generationally transmitted, suggesting a powerful potential way of maintenance inheritance of the effects of early chronic stress [8]. Like sperm cells, oocytes may also carry epigenetic anomalies resulting from stress exposure since the trans-generational inheritance of stress-induced symptoms occurs through females independently of maternal care [107].

The interaction between genes and the environment is necessary for the animals’ life, and environmental enrichment changes the epigenetic nature of an organism, enhancing neural plasticity, resilience to stressors, and repair [108]. After more than six decades of work on environmental enrichment, we laud the advances in understanding relevant biological parameters and critical mechanisms; however, further research will have to identify the stage of life and how long it takes enrichment to activate benefits, promote plasticity and improve resilience.

## 5. Neural Mechanisms Related to Stress Resilience

The central nervous system (CNS) anticipates present and future needs based on experience. By having corrected errors, the CNS learns how to prevent them [109]. Without a CNS, the organisms’ feedbacks concerning environmental variations would be limited to ensuring biochemical homeostasis. The CNS allows adaptation in response to a set of predictable and unpredictable conditions without a set reference. When an organism perceives a critical change or threat as being stressful, the amygdala, throughout the hypothalamus, activates the Sympathetic-Adrenal-Medullary System (SAS) to cope with changes, by the so-called “flight or fight” response. This response, immediate to stress but usually of relatively short duration, elicits a range of autonomic responses and the release of catecholamines into the bloodstream affecting different biological systems (cardiovascular, respiratory, gastrointestinal) to provide more energy to the body necessary to face a threat or to escape from it. The HPA axis is the second to intervene by stimulating the adrenal cortex to release glucocorticoids, triggering the metabolism for the maintenance or reinstatement of homeostasis during stress.

**Figure 9:**
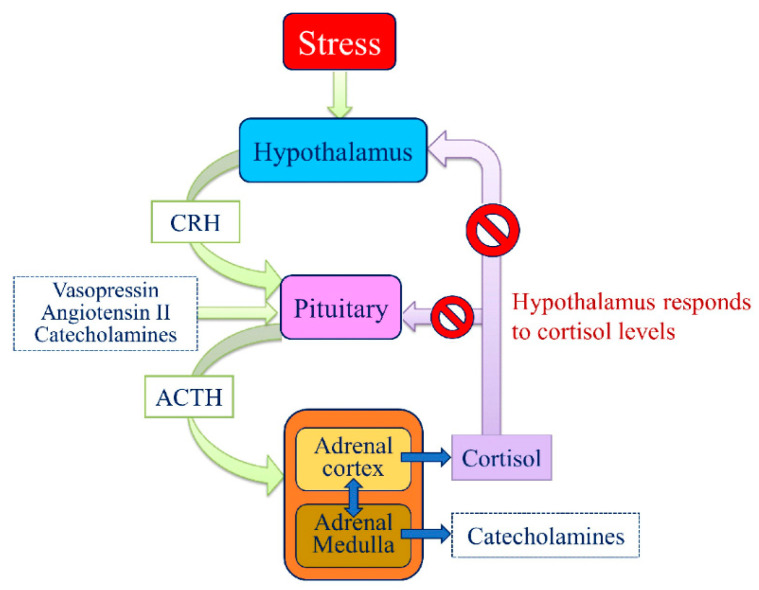
Schematic overview of the hypothalamic-pituitary-adrenal (HPA) axis. Stress activates the secretion into the portal circulation of the corticotropin-releasing hormone (CRH) and other ACTH secretagogues (e.g., vasopressin) then, ACTH stimulates the adrenal glands to synthesize and releases norepinephrine and glucocorticoids, primarily cortisol. Glucocorticoids, in turn, act back on the hypothalamus and pituitary (to suppress CRH and ACTH production) in a negative feedback cycle.

Once the disturbance has normalized, these two primary systems return to baseline due to negative feedbacks in the CNS. Indeed, the physiological stress response is noteworthy more complex, involving several other systems. Among these, the medial prefrontal cortex (mPFC) and the limbic system play a pivotal role by directly or indirectly responding to stress, exerting their effects on the hypothalamus, the pituitary gland, and the adrenal gland of the HPA axis (Figure 9).

The HPA axis, together with the SAS and the Immune System (by hormonal and neural communication), constitutes the stress system, whose adjustments contribute to increase or decrease the resilient response [110,111]. This complexity explains why even if the responses the animals elicit to deal with a stressful situation are integrated and coordinated at the CNS level, their expression greatly varies in each individual, according to the individual genetic background, life history, personality, environmental and social context and the component of novelty, predictability and controllability of the stimulus itself.

The CNS carries out its resilience activity to environmental stresses by modulating the length and branching of dendrites, and this neuroplastic activity is epigenetically controlled. In this sense and with the neuroplasticity maintained throughout life, we can consider the CNS an organ capable of producing resilience through allostatic responses mediated by hormonal, inflammatory, and metabolic molecules [112].

The brain is the central stress adaptation organ in as much as it perceives and determines appropriate behavioral and physiological responses. Brain structure is dynamic and in continuous evolution; indeed, after stressful experiences, the brain changes architecture and functions through internal neurobiological mechanisms. In mammals, brain architecture shows plasticity throughout adult life, and neuroepigenetic studies reveal a dynamic and ever-changing brain. A key feature of neuroepigenetic marking is that it is stable, sometimes across cell generations, and reversible. In the presence of new challenges, epigenetic mechanisms will characterize vulnerability or resilience to future stressors. The stimulatory and inhibitory neurochemical circuitry creates a physiological organization that modulates and finely-tunes the adaptive stress response within the CNS. Adaptive physiological change neutralizes the “good” stress factors, but should these be prolonged; resilience is the rheostat that regulates the well-being of the CNS [113].

Chronic stress can be the source of harmful neurocognitive effects, particularly in the hippocampal region. The hippocampus is mainly a plastic/elastic and vulnerable structure in the medial temporal lobe, implicated in consolidating memory in humans and spatial memory in rodents and a target of stress hormones [114]. Many studies on animal models demonstrated structural hippocampal plasticity, especially during the progressing neurogenesis of the dentate gyrus (DG) [114] and in the remodeling of dendrites and synapses in the critical neurons in the Ammon horn [114]. Hippocampal neurons express receptors for circulating adrenal steroids [115]. There are mainly two types of adrenal steroid receptors: type I (for mineralocorticoids) and type II (for glucocorticoids), mediating a variety of effects on neuronal excitability, neurochemistry, and structural plasticity [114]. In the presence of stressful experiences, steroids, and excitatory amino acids, neurotransmitters (principally glutamate and aspartate) participate in neurogenesis regulation. Jankord et al. [116], have shown that the stimulation of the hippocampus reduces glucocorticoid secretion, while its lesion causes increasing basal levels of glucocorticoids, especially during the stress recovery phase. Through negative feedback mediated by glucocorticoids on the HPA axis, the hippocampus partakes in the termination of a stress response. Numerous studies conducted by Lupien et al. [117] show that stress and stress hormone exposure impairs hippocampal-dependent memory systems in humans and animals. Early life stress altered hippocampal plasticity and contributed to memory impairments associated with stress [117,118].

**Figure 10:**
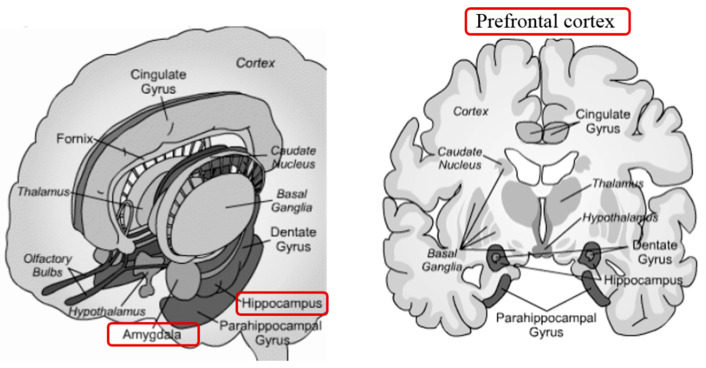
The limbic system is the portion of the brain, which deals with stimulation, arousal, emotions, and memories.

Another brain region involved in the mediation of stress-related behaviors and hippocampal modulation is the amygdala. The amygdala is one main structure of the limbic system (Figure 10).

It mediates sensory inputs from different brain areas and projects towards various autonomic and somatomotor structures, intermediating defensive responses—suggesting that the amygdala may also be involved in the mediation of the effects of stress on CNS plasticity and hippocampal memory. A rat model study revealed that amygdala lesions showed the effects of stress on hippocampal plasticity and memory. Physiologically, hippocampal slices from stressed rats with amygdala lesions exhibited normal long-term potentiation (LTP). On a behavioral level, spatial memory remained intact in animals with stress-induced amygdala lesions [119]. While acute stress rapidly alters neuronal connectivity in the hippocampus and medial prefrontal cortex (mPFC) [8], chronic stress represses the HPA axis mainly through the inhibitory projections of the ventral prelimbic cortex (PLC), infralimbic (IC), and anterior cingulate (ACC) that target the neurons of the HPA axis directly or indirectly through the relays in the adjacent brain regions [120].

The prefrontal cortex (mPFC), particularly in its ventromedial portion (vmPFC), mainly reacts to stress (fear, decisions) and therefore has an active role in managing emotions. It receives inputs from the ventral tegmental region (VTA; motivation, knowledge), from the amygdala (emotional values), from the temporal lobe (affectivity, relationships, instinctive reactions and behaviors, visual recognition, auditory perception, and memory), from the olfactory system (olfactory perceptions), and the thalamus (motor and sensory coordination). In turn, mPFC sends its signals to the temporal lobe, the amygdala, the hypothalamus, the hippocampus, and the cingulate cortex: by its cross-talking with the hippocampus and amygdala, it represents a key area to deal with allostatic loads and to develop resilience. An essential feature of the ventral mPFC is its role in acquiring stress resilience, particularly in experience-driven resilience involving progressive coping response learning. In animals, resilience can be gained by exposure to a controllable stress factor (i.e., tail shock). The acquired resilience is long-lasting; it is mediated by pyramidal glutamatergic cells in the ventral mPFC, which act as controllability detectors. Stress resilience can also be acquired by previous exposure to an enriched environment, but, in this case, it involves the infralimbic cortex, the hypothalamus, the dorsal raphe nucleus, or the amygdala [8].

As within the hippocampus, chronic stress causes narrowing of dendrites, loss of “crown of thorns” in the medial prefrontal cortex, and expansion of dendrites in the orbitofrontal cortex (OFC) and PFC [121]. When the stress is finite, the remodeled brain circuits recover, at least in younger animals, with healthy brain architecture. However, studies on gene expression demonstrate that the recovered state was not equal to the initial state, influencing responsiveness to subsequent stress factors: these changes in transcriptome reactivity represent a molecular signature for resilience [114].

Recent studies have focused their attention on optogenetics: an emerging science that combines optical and genetic detection techniques, in order to probe neuronal circuits inside intact brains of mammals and other animals for periods of milliseconds, the time needed to understand how information is processed and handled between neurons. Rat animal model of early-life stress appears to affect a substantial on HPA axis gene expression levels. Fast early handling (brief separation) is associated with long-term hippocampal glucocorticoid receptors (GR) overexpression and stress reduction. Conversely, prolonged maternal separation caused a vulnerable phenotype, with a low GR mRNA level and prolonged neuroendocrine response to stress [122]. Furthermore, research into early experiences revealed that intermittent early maternal separations promote resilience by increasing cortical volume in ventromedial PFC (VMPFC). Direct optogenetic stimulation of mPFC neurons, with channelrhodopsin (ChR2), promoted resilience to stress from social defeat [123].

Susceptibility to chronic social defeat stress is associated with an increasing glutamatergic tone, including a higher frequency of excitatory currents and some glutamatergic synapses, on medium spinous neurons in nucleus accumbens (NAc); altogether, recent evidence showed that resilience is partly mediated by an active adaptation that counteracts this susceptibility mechanism [124]. To date, molecular research in resilient mice exposed to chronic stress or social defeat is linked to mRNA overexpression of Fos family transcription factors (c-Fos, FosB, Fra-1, and Fra-2) mPFC glutamatergic neurons. Fos family genes are involved in critical cellular events, including cell proliferation, differentiation, cell survival, and the early overexpression of these gene families, suggesting an mPFC neuronal activation as a pro-resilience adaptation. A prominent pro-resilience marker is the transcription factor FosB, which is activated persistently by neuronal activity. In NAc, the basal FosB expression can predict whether a mouse will be resistant or sensitive to stress by social defeat: FosB high expression is related to resilience while the low expression to susceptibility.

Furthermore, the induction of delta FosB (ΔFosB) in NAc is necessary and sufficient for causing stress resilience: its overexpression blocks isolation-induced stress vulnerability and acts as an antidepressant, while its inhibition promotes susceptibility. The transcription factor FosB can regulate the expression of multiple genes, including metabotropic Glutamate Receptor 2 (GluR2). In resilient mice, medium spiny neurons GluR2 expression increases after chronic social defeat, reducing neuronal excitability and weakening the stimulation of NAc by glutamatergic input. Conversely, in sensitive animals, GluR2 expression decreases, and consequently, neuronal excitability increases, stimulating glutamatergic NAc. The glutamatergic input of NAc can promote or prevent motivated behaviors associated with resilience and vulnerability. It is unclear how FosB is regulated in NAc, but transcriptional changes probably occur via the serum response factor (SRF) activating cellular immediate-early gene response (IEG). Vulnerable mice showed SRF downregulation in NAc, as demonstrated in depressed patients [8].

## 6. Concluding Remarks

This review dealt with resilience through the analysis of environmental stressors and molecular and phenotype changes occurring in resistant subjects. Interactions with the environment expose the animal to both beneficial and threatening situations. The perception of animals to environmental stimuli is influenced by historical events, the current state of well-being, and cognitive or behavioral capacity, and the outcome of epigenetic changes may be resilience or vulnerability. Understanding the link between the observed environmental stressor, the epigenetic mechanism, and the associated final event will lead to a better understanding of the mechanisms underlying individual differences in resilience.

This review was written to introduce the reader to the concept of resilience as an acquired and developed ability throughout life, not only inherited at birth. The nervous system, as well as other systems, are highly dynamic structures that show plasticity throughout life and, having the opportunity to act on this plasticity to modify resilience to stressors could be an asset not only to improve animal welfare but also to increase livestock productivity or the quality of the food derived from them. Epigenetic changes that are meiotically and mitotically inheritable can improve resilience and, therefore, animal welfare. Understanding how to act to epigenetically reprogram resilience could be a great gift for the next generation. We would like to conclude this review by mentioning an epigenetics dogma that summarises our thoughts “genes learn from experience” [125]; however, the exact nature of the interaction between the genes and the environment, which is targeted by the paradigm, remains elusive to this day. 

## Data Availability

Not applicable.
